# Beyond classification: how preschool children select attributes and construct variables in data modeling

**DOI:** 10.3389/fpsyg.2026.1996058

**Published:** 2026-10-21

**Authors:** Esra Ay-Karaçuha, Neslihan Şahin

**Affiliations:** 1 Ministry of National Education of the Republic of Türkiye, Samsun, Türkiye; 2 Department of Mathematics Education, Faculty of Education, Sinop University, Sinop, Türkiye

**Keywords:** attribute selection, category construction, data modeling, early childhood education, multiple-case study, preschool children, statistical reasoning

## Abstract

**Introduction:**

Preschool children are known to notice multiple attributes in the same objects, but far less is known about how they select one of these as an organizing criterion and turn it into a category structure. This study examined which attributes preschool children noticed in an unstructured data set, which they selected as an organizing criterion, how they justified that selection, and how they formed, negotiated, and revised category structures as they applied the selected attribute to the data set.

**Methods:**

In this qualitative multiple-case study, two focal groups (*n* = 4 each), drawn from the 11 children (mean age 5 years 8 months) in a four-week implementation in a Turkish public kindergarten, were each treated as a case. The analysis centered on one activity using museum-themed data cards with no predefined categories. Video recordings, transcripts, observation notes, and children’s products were analyzed within and across cases.

**Results:**

The children noticed several attributes in the same data units, including object type, color, and material, but not every noticed attribute was used to organize the data. Within-group negotiation shaped attribute selection, and the groups structured the same data set using different criteria. They also differed in sustaining the chosen criterion: one applied it consistently across the data set, whereas the other revisited it. While building categories, they evaluated object–category fit, justified their decisions, and returned to the criterion when a case did not fit.

**Discussion:**

The findings suggest that constructing a variable may be less a single moment of selection than an iterative process of noticing attributes, negotiating a criterion, and testing the resulting structure, making pre-representational decision processes in early data modeling visible through the lens of attribute structuring.

## 1 Introduction

Data has become a basic component of decision-making, problem-solving, and sense-making in contemporary society (English, 2018). As digital technologies have become widespread, individuals encounter increasingly large and complex data sets, which require not only access to data but also the ability to select appropriate data, identify relationships among them, and draw meaningful inferences from those relationships (Paparistodemou et al., 2021). Statistics education research has therefore emphasized in recent years that thinking with data should be treated not as a topic of statistics instruction alone, but as a fundamental mode of thinking to be developed from an early age (Bargagliotti et al., 2020; Garfield and Ben-Zvi, 2008; Lewis Presser et al., 2022; Paparistodemou et al., 2021).

In early childhood, children naturally compare the objects and events around them, notice similarities and differences, form classifications, and use those classifications to make sense of their environment; these processes are regarded as early forms of statistical reasoning (English, 2014; Lehrer and Schauble, 2004). Early statistical thinking, however, is not limited to counting or grouping objects. Distinguishing the data relevant to a problem situation, deciding which attributes are meaningful for that problem, and using those attributes to organize data are all considered critical cognitive processes in the development of statistical thinking (Leavy and Hourigan, 2018, 2021; Mulligan et al., 2024; Reading and Shaughnessy, 2004; Zapata-Cardona, 2023).

The data modeling approach offers an important theoretical framework for examining these processes. Data modeling is defined as an iterative problem-solving process that involves examining data to address real-life problems, identifying appropriate attributes, organizing the data, and developing models that explain the problem situation (English, 2012, 2014; Lehrer and English, 2018). Its aim is not merely to produce a graph or a table; it is to identify meaningful variables that explain the problem situation and to structure the relationships among them (Lehrer and Schauble, 2004).

One of the most important components of this process is evaluating the attributes in a data set within the problem context. Children can notice many attributes in a data set at once, but not all are equally meaningful for solving the problem. Children therefore first need to notice potential attributes in the data set, then select those that fit the problem context, and finally use them to organize the data. Lehrer and Schauble (2004) argue that variables are not already present in data sets; rather, the attributes that individuals find meaningful within the problem context are structured as variables. Similarly, Leavy and Hourigan (2018) showed that in selecting attributes, preschool children drew not only on perceptual similarities but also on contextual relations. Recent studies likewise show that children’s processes of selecting and organizing attributes when working with data constitute a central area of research for understanding the development of statistical reasoning (Dvir and Ben-Zvi, 2023; Kinnear, 2023; Zapata-Cardona, 2023).

Much of the research in this area has focused on preschool children’s skills in collecting data, constructing representations, or interpreting graphs (English, 2012, 2014; Leavy and Hourigan, 2018; Paparistodemou et al., 2021). By contrast, it remains insufficiently explained which attributes children notice when they encounter unstructured data sets, which attributes they choose to use to solve the problem, and how these decisions relate to processes of emergent variable construction. Studies published in recent years emphasize, in particular, that data modeling research should focus not only on the representational products that emerge but also on the cognitive decision processes that direct children’s organization of data (Dvir and Ben-Zvi, 2023; Fry et al., 2024; Mulligan et al., 2024). This orientation indicates that work on data modeling in early childhood shifts from the question “which representation did the children construct?” to the question “which attributes did the children select, why, and how did they structure these selections?”

Building on this theoretical gap, the present study examines how preschool children notice attributes in data modeling activities, which attributes they select as organizing criteria, and how they structure data organization based on these selections. Group interactions from data modeling activities conducted in a natural classroom setting were analyzed using a cross-case approach to identify commonalities and differences in groups’ processes for identifying and selecting attributes. In this respect, the study offers the early childhood data modeling literature that accounts not only for the categories children construct, but also for the cognitive processes through which those categories emerge.

### 1.1 Theoretical foundations of the study

This study’s theoretical basis lies at the intersection of approaches to early statistical reasoning, data modeling, context-sensitive attribute selection, and the categorical structuring of data sets. Children’s interactions with data are treated here not as a simple sorting activity in which they apply predetermined categories, but as a process of meaning-making: they notice the possible attributes in a data set, evaluate how well those attributes fit the problem context, negotiate alternative criteria within the group, and give the data set a particular structure through the criteria they select. The focus of the study, then, is less on the final products children construct than on how the attribute decisions that make data organization possible emerge and stabilize through group interaction.

### 1.2 Data-based thinking and statistical reasoning in early childhood

Statistical reasoning cannot be reduced to the ability to obtain numerical results or to read information from ready-made graphs. Understanding a problem that concerns data, determining which knowledge is relevant to it, evaluating the similarities and differences in the data, organizing those differences according to meaningful criteria, and explaining the resulting structure within its context are likewise among its basic components (Garfield and Ben-Zvi, 2008; Wild and Pfannkuch, 1999). Statistical thinking thus involves decisions about what counts as data and which attributes of the data are worth examining—decisions that precede any technical operation performed on the data.

In their everyday experiences, children in early childhood compare objects, notice similarities and differences, form temporary groups, and reorganize those groups as situations arise. Although none of these activities can be called formal statistics, together they provide important cognitive resources that support children’s later data-based thinking. The GAISE II framework likewise emphasizes that statistical investigation at the earliest level requires children to encounter meaningful questions, identify data appropriate to the question, organize the data, and relate the resulting structure to the context (Bargagliotti et al., 2020). The development of early statistical reasoning is therefore connected not only to children answering the question “how many?” but also to their reflection on questions such as “which objects can be considered together?” “What is the attribute that allows us to think of these objects together?,” and “How would the data structure change if a different criterion were selected?”

Current research shows that young children can display more complex forms of statistical thinking than expected when given developmentally appropriate, contextually meaningful tasks. Kurt (2023), for example, found that young children could discuss probabilities in terms of the sample space, use language that expresses uncertainty, and develop informal data-based inferences. Kinnear (2023) showed that the ways children interpret a data structure are influenced not only by the numerical values displayed but also by the data context and by the expectations the task creates. These studies indicate that early statistical thinking does not develop as a set of context-independent skills; children’s decisions about data are shaped by the problem situation, their prior experiences, and how the task is framed.

In investigating how children think with data, it is therefore not sufficient to focus only on whether classification outcomes are correct or incorrect. Which attribute a child notices, which attribute the child treats as important, how the child accounts for that selection, and how the child revises a decision in the face of other children’s suggestions should also be treated as observable components of early statistical reasoning. This approach makes it possible to understand children’s thinking not in terms of fixed competencies but as processes that develop within context and are reshaped through social interaction.

### 1.3 Data modeling: a cyclical process of meaning making

One of the principal theoretical foundations of this study is the data modeling approach. Data modeling does not assume that the data relating to a problem situation are “given” in any direct or neutral sense. It instead treats as open to question which phenomena will be taken as data, which attributes will be recorded, how those attributes will be organized, and how far the resulting data structure can account for the problem situation (English, 2010, 2012; Lehrer and Schauble, 2004). Data modeling in this sense involves not merely processing data but constructing a data structure meaningful to the problem context.

English’s studies of data modeling in the early years show that children move back and forth among the processes of making sense of the problem situation, examining data sources, identifying the attributes they consider important, organizing the data according to those attributes, and reviewing the model they have developed (English, 2010, 2012, 2013). This process is not linear. Children may notice that a criterion they selected at the outset cannot be applied to every object in the data set, may begin to try a different attribute, or may reorganize categories they had formed earlier in line with their peers’ suggestions. Movement from one phase of the cycle to another should therefore be understood not as completed, irreversible progress, but as an iterative process in which the model’s fit to the problem situation is continually tested.

Recent literature more explicitly shows that data modeling cannot be defined solely by producing a particular type of representation. Noll et al. (2022) approach statistical modeling through the dynamic relations established among data, context, and uncertainty, and note that modeling is not a fixed sequence of operations applied to data but a process in which meaning about the problem is continually reconstructed. Dvir and Ben-Zvi (2023) likewise conceptualize informal statistical models as structures of meaning that learners develop, use, and, when necessary, revise as they connect data with context. On this view, a model is not only a final product: children’s decisions to classify and reclassify data are themselves part of the modeling activity.

Similarly, Mulligan et al. (2024) showed the importance of a pedagogical cycle that connects the phases of problem posing, data generation, data organization, interpretation, and data-based reasoning in data modeling activities conducted in the first years of primary school. Classroom communication that lets children try different solutions and reconsider earlier decisions also appears decisive in developing their thinking about data. Fry et al. (2024) show that children’s understandings of data are “cognitively tuned” through explanations, counter-suggestions, and joint decisions in the classroom. That change occurs not through individual discovery alone, but through reshaping ideas in communication.

While this study takes English’s cyclical approach as its basis, it focuses in particular on the decision processes that occur before data are structured categorically. Rather than reconfirming English’s modeling cycle, it aims to specify how attribute identification and data organization—which often appear as a single phase within that cycle—develop independently. When children look at a data set, they notice more than one possible attribute, select one, discuss their selections with their peers, sometimes revise their criteria, and finally reach agreement on a particular categorical structure; these actions constitute a distinct micro-process within the data modeling cycle.

### 1.4 The relationship among attribute, variable, and category

An attribute in a data set is a property or quality used to describe the units of observation. Color, shape, material, purpose of use, and object type are examples of attributes that might be considered in a given data set. Not every observable property becomes a variable of the study on its own, however. For an attribute to function as a variable, it must be related to the problem situation, recognized as capable of taking different values across the units of observation, and used as a consistent criterion in organizing the data.

The concepts of attribute, variable, and category are therefore related but not equivalent. An attribute refers to a property of an object or phenomenon that can be attended to; a variable refers to the dimension of that property which differs across the units in a data set and is structured for inquiry; and a category refers to one of the qualitative values a variable can take, or to one of the groups formed based on those values. In a data set of historical artifact cards, for example, “material” is a possible attribute and candidate variable, whereas “gold,” “silver,” and “iron” are categories or values it can take. When children approach the same cards through the attribute of “object type,” by contrast, they may form different categories such as “coin,” “jewelry,” “vase,” and “statue.” The same data set can thus be turned into different data structures depending on the attribute selected.

Lehrer and Schauble’s modeling approach shows that when variables are not presented to children as ready-made labels, children’s determining which attributes are meaningful for the problem is a basic component of statistical modeling (Lehrer and Schauble, 2004). On this view, constructing a variable is not merely naming an attribute already present in the objects: the attribute must be made comparable, traced across the entire data set, and used as the basic criterion for organizing the data. Children’s naming of differences such as “gold,” “silver,” or “brown” may therefore indicate attribute awareness; turning color into a shared criterion that organizes the whole data set, however, represents a further step toward variable construction.

Leavy and Hourigan (2018) study with 5 to 6-year-old children is particularly important with respect to this distinction. The study found that perceptual similarities influenced children’s initial attribute selections, but that the problem situation, knowledge about the data context, and within-group negotiation could direct children toward more functional and contextual criteria. The children did not remain bound to the attribute they noticed first; they moved to attributes more closely related to the problem—taxonomy, habitat, behavior, and diet—and revised and expanded their categories. This finding shows that attribute selection is not a one-time individual decision but a process shaped jointly by perceptual attention, contextual knowledge, and social interaction.

### 1.5 Context-sensitive attribute selection

Attribute selection is one of the most fundamental yet least visible decisions in the data modeling process. When the variable headings of a ready-made table are defined in advance, the decision has already been made for the learner. In unstructured data sets, or in data sets open to multiple solutions, children must decide for themselves which attribute to examine. Attribute selection then becomes not simply an operation of visual discrimination but an interpretation of what the problem situation is about.

Current research on early statistics education clearly emphasizes the role of context in this process. Kinnear (2023) showed that children can interpret the same data values differently across data and task contexts, suggesting that meaning about data does not arise from numerical or visual structure alone but is produced alongside the contextual expectations children hold about the data. Frischemeier and Schnell (2023) likewise show that contextual expectations can direct data analysis in statistical investigations at the primary level, and that as children examine data they establish relations between the data and the context-based assumptions they have formed earlier.

Zapata-Cardona (2023) showed that presenting data modeling tasks within cultural story contexts meaningful to children can support early statistical reasoning about organizing, structuring, and making sense of data. In that study, context functioned not as a decorative element that merely increased children’s interest, but as an epistemic resource affecting which data would be seen as important and which relations would be established among them. Children’s attribute selections, then, cannot be inferred from the visual properties of the data cards alone; the task’s aim, the problem narrative, and children’s knowledge of the context must also be taken into account.

Context can also influence how children conceptualize the same object in different ways. An object may be treated as “gold” in terms of its visible color, as “metal” in terms of the material it is thought to be made of, and as a “coin” or a piece of “jewelry” in terms of its use or type. These labels are not merely different words; they represent alternative attribute structures that could organize the data set in different ways. Children’s selection among these possibilities depends as much on which attribute is more visible as on which attribute is found more explanatory within the problem context.

For this reason, the present study does not treat perceptual and conceptual/contextual attribute selection as mutually exclusive developmental levels. Selecting a visually salient attribute does not necessarily indicate superficial thinking, nor does selecting a functional attribute indicate more advanced thinking in every case. The meaning of a selection must be evaluated together with the criterion’s relation to the aim of the problem, how consistently it can be applied to the data set, and how the child justifies that selection.

### 1.6 Attribute structuring: the analytic lens of the study

In this study, attribute structuring serves as a study-specific analytic lens describing how children select a classification criterion from among the attributes they have noticed, form category boundaries appropriate to that criterion, negotiate objects’ category memberships, and, when necessary, reorganize their criteria or categories. The concept is not proposed as an established, completed developmental model in the literature; instead, it is derived from a theoretical synthesis of English (2013) cyclical data modeling approach, Lehrer and Schauble (2004) views on structuring variables, Leavy and Hourigan (2018) findings on attribute selection, and current context-oriented research.

The process involves several related but analytically distinct moves. First, children notice attributes in the data set that could serve as classification candidates; at this stage, they may voice more than one attribute—object type, color, material, or function—at the same time. Second, select one candidate attribute as the criterion for organizing the data. Third, determine the category values of the selected attribute and match objects to those categories. Fourth, test the applicability of the selected criterion to the data set: when children notice that an object does not fit easily into any existing category, they may redefine the category boundaries, form a new category, or abandon the previous criterion and move to a different attribute. The final move is the group’s provisional agreement on a particular criterion and category structure.

Revising a criterion should not be interpreted merely as indecision or failure. Noticing that a categorization based on one attribute does not work across the entire data set, and trying another attribute, may indicate that children are testing the adequacy of the data structure they have constructed. From the perspective of Dvir and Ben-Zvi (2023) approach to informal statistical models, such reorganizations can be seen as the evaluation and revision of the model in light of data and context; similarly, returns within the data modeling cycle can be interpreted as an early form of model evaluation concerning whether the structure constructed serves the aim of the problem.

Justifying category membership is likewise an important component of attribute structuring. A child placing an object in a particular group and explaining why it was placed there does not carry the same analytic meaning: an explanation makes visible the knowledge on which the child bases the category boundary. The absence of an explanation, however, does not necessarily mean the decision was arbitrary, since young children may apply the criterion they use consistently in their actions while having difficulty expressing it verbally. Children’s verbal explanations, their card placements, their responses to peers’ suggestions, and changes in the classification arrangement should therefore be evaluated together.

Within-group negotiation extends this process beyond individual noticing. Fry et al. (2024) findings on classroom communication show that children’s understandings of data can be reshaped when they encounter others’ explanations. While one child proposes a perceptual criterion, another may offer an alternative based on object type or purpose. As a result, the group may retain the initial proposal, shift to a different criterion, or recognize that more than one valid organization exists for the same data set. Social interaction thus supports not only sharing a decision but also testing possible variables and jointly constructing category structures.

### 1.7 The place of variation in attribute structuring

Variation is one of the central concepts of statistical thinking, because the rationale for thinking with data arises from the fact that observations are not all the same and that these differences require explanation (Wild and Pfannkuch, 1999). In the early childhood context, however, it is not sufficient to claim variation based on children’s use of the word “different” or their separation of objects into different groups. What must be examined is the attribute by which children see difference, whether they trace that difference across the entire data set, and whether they use it as a consistent criterion for organizing the data.

Lehrer and English (2018) emphasize that introducing children to variation requires thinking about the data as an aggregate rather than about individual values. In this study, however, variation is not treated as the spread of a distribution or as a formal statistical measure. Given the age level and data structure of the study, it is examined primarily in terms of noticing that data units differ with respect to particular attributes and organizing that difference within a categorical structure.

Noticing an attribute may in this respect offer an early point of departure for variation, but awareness of variation and naming an attribute are not entirely synonymous. A child stating that there are “gold,” “silver,” and “brown” objects shows that the child has noticed difference with respect to color; using color as a shared criterion applied to the entire data set shows that this difference has been structured. Recognizing that the same data set could also be organized by object type or material may indicate a more flexible understanding that variation can produce different data structures depending on the attribute.

Buehring and Grando (2023) study with children on multivariate data shows that young children can participate in data situations involving more than one variable, and that such processes can provide an opportunity to read and explain the world through data. This result suggests that children noticing more than one possible attribute in the same data set should not be regarded merely as a distraction; it may be an important resource for seeing that a data set can be restructured according to different questions. Whether children can sustain these possibilities simultaneously or construct nested variable structures, however, is a more complex capability that requires separate investigation.

This study does not claim to investigate children’s formal thinking about distribution or spread. On a more limited reading appropriate to the data, it examines the early foundations of variation through children’s linking differences to particular attributes, using those attributes to form categories, and reorganizing the same data set with alternative criteria. Variation is thus not equated with classification itself; it relates to noticing and structuring the differences that make classification possible.

### 1.8 The integrative theoretical framework of the study

This study’s theoretical framework rests on the assumption that children are not passive processors of data who use ready-made variables in the data modeling process. Children notice possible attributes in a data set, evaluate their suitability for the problem context, negotiate alternative criteria with peers, and form category structures based on the criterion they select. When these structures prove inadequate or inapplicable, they are able to revise the criterion, redefine category boundaries, and review their data organization.

The dimensions of the process examined along these lines are children’s noticing attributes, selecting the attribute related to the problem, forming category values, negotiating category membership, testing the fit of the criterion to the data set, reorganizing the criterion or the categories, and provisionally stabilizing a particular attribute structure.

These moves are not rigid, linear phases occurring in the same order in every group; they are analytic processes through which children may move cyclically as they interact with data and context. The aim of the study is not to show once again that five-year-old children can classify, but to account in greater detail for this part of the data modeling cycle by making visible the moves of noticing, selecting, negotiating, testing, and reorganizing attributes that occur before a classification system emerges.

In line with this framework, the study focuses on three main questions:

1. Which attributes do children notice as candidate classification criteria in an unstructured data set?
2. Which of these attributes do they select, and how do they justify their selections?
3. How do they form, negotiate, and, when necessary, revise category structures as they apply the selected attribute to the data set?

Through these questions, the study aims to account for data modeling in early childhood by examining the data-structuring decisions that occur before final products.

## 2 Method

### 2.1 Research design

This qualitative case study examines how preschool children notice and select attributes and how they use them to organize data during data modeling activities. Its central aim is not to evaluate the final products children construct, but to understand the cognitive and social reasoning processes that emerge during their interactions with data. The study was therefore designed as a multiple-case study, an approach that allows researchers to examine interactions in a natural learning environment holistically (Stake, 2006; Yin, 2018).

Case studies allow researchers to examine a phenomenon in detail within its natural context and compare cases (Merriam and Tisdell, 2016; Yin, 2018). In this research, each group of children was treated as an independent case, and a cross-case analysis was then conducted to reveal common patterns and diverging data organization processes across the groups (Stake, 2006). This made it possible to examine not only the characteristics of a single group, but also the different ways groups structured a similar problem situation. In this study, the case is how the two focal groups dealt with the attributes of the data—relating the data cards to the context of the activity—as they worked on the museum activity.

The unit of analysis is not individual children but the data modeling processes developed jointly within the group, because data modeling activities make visible the shared reasoning processes that take shape during group discussion rather than children’s individual decisions. This approach also aligns with qualitative educational research that holds that early childhood learning is constructed through social interaction (Merriam and Tisdell, 2016).

### 2.2 Participants and research setting

The research was conducted in a kindergarten class at a public primary school in Türkiye. The participants were 11 children—six girls and five boys—with a mean age of 5 years 8 months. None of the children had attended any preschool institution before the study, so the data modeling activities constituted their first systematic experience of data modeling in preschool education.

The teacher of the class in which the research was conducted is also one of the researchers. The implementation group was selected from the class taught by that researcher to preserve the natural flow of the extended implementation, to avoid disrupting classroom routines, and to keep the children from being affected by the presence of an unfamiliar researcher; in qualitative educational research, conducting a study within its natural context allows participants’ everyday learning processes to be observed more realistically (Cohen et al., 2018; Merriam and Tisdell, 2016). This dual role nonetheless carries two risks: children may shape their contributions toward what they believe their teacher expects, and the researcher who designed and taught the activities may be influenced by prior expectations when interpreting the data. Three measures were taken to address these risks.

First, the teacher-researcher’s role during the implementation was limited to guidance. The children were not told which attribute to select, how to organize the data, or which categories to form; when they were unable to proceed, the process was supported with open-ended questions. During this process, the teacher guided the children by asking questions such as “What attributes are there?” and “According to which attribute did you group them?,” helping them notice attributes, justify their classification decisions, and engage in discussions about the data structuring process. When an open-ended question was not understood, the question was narrowed to what the children were attending to at that moment (for example, “What is it made of?”). Because the activities and data cards were designed as educational tools, this limited guidance is part of the implementation; however, we acknowledge that such questions may direct children’s attention toward a particular attribute. Second, two researchers coded independently, one of whom was not the class teacher, and then compared the codes and discussed divergent interpretations. Third, moments in which a researcher’s question may have directed the children’s attention toward particular attributes were not excluded from the analysis; these moments are stated explicitly in the findings, and naming an attribute was treated as analytically distinct from selecting that attribute as the criterion for organizing the data.

### 2.3 Data modeling activities and the implementation process

The researchers developed the data modeling activities used in the research. Their design was based on the beginning-level statistical investigation skills defined in the GAISE II report (Bargagliotti et al., 2020), the NCTM data analysis standards (NCTM, 2014), and English (2010, 2012, 2014) approach to data modeling in early childhood.

In line with English’s approach, the activities were designed as real-life problems in which children could construct their own data organizations rather than work with ready-made variables. The authors took particular care to ensure that the problem situations did not contain a single correct solution path, allowed for different data organizations, and presented open-ended tasks in which children could develop alternative attributes (English, 2012, 2014).

The authors developed four data modeling activities and implemented one each week over 4 weeks (Table 1). Before implementation, three experts in mathematics education and mathematical modeling evaluated the activities, and the authors made the necessary adjustments based on their feedback. Each activity consisted of three sessions (Figure 1) of approximately 50 minutes each, held on Monday, Tuesday, and Wednesday, so the research was carried out over twelve implementation sessions.

**Table 1:** Data modeling activities, task contexts, cognitive/theoretical focus, and targeted skills.

Activity	Task	Cognitive/theoretical focus	Targeted skills
Shoe activity (18 cards)	How can we organize shoes in a women’s shoe store by their features?	Develop alternative classification criteria and transform the data into a structured grid representation. This activity focuses on the initial stage of data modeling: organizing data according to attributes and creating a representation.	Working with the cards depicting women’s shoes: Identifying and selecting attributes, noticing variability, categorizing the data according to its attributes, positioning the data cards on the grid, collaboratively discovering the basic components of a graph through the grid, creating a representation/model on the grid, transitioning from the grid to the graph and constructing a picture graph on structured graph paper, reading and interpreting the graph.
Museum activity (26 cards)	How can we exhibit the historical artifacts uncovered in an archaeological excavation site?	Organizing historical artifacts according to their different attributes and making decisions about how to exhibit them, restructuring the representation, and transitioning from the grid to the graph. This activity focuses on the relationship between data organization and individual re-representation.	Working with the cards depicting historical artifacts exhibited in Turkish museums: Organizing the data, defining attributes, categorizing the data, displaying the data on the grid, reading the data from the grid, transitioning from the grid to the graph, transforming the data cards categorized on the grid into individual graphical representations, restructuring the representations, reading and interpreting the graph.
Picnic activity (30 cards)	Which type of waste do you think is most common in the picnic area? How can we find the answer?	Comparing predictions to data within the context of environment and recycling, noticing most-least relationships, and making sense of data through representation. This activity focuses on children’s ability to relate the data organization process to prediction and interpretation.	Identifying attributes, classifying waste types, categorizing the data, arranging the data on the grid, noticing most-least relationships, comparing predictions to the data, representing the data graphically, reading and interpreting the graph.
Toy activity (45 cards)	How can we place the newly requested toys into the play centers of our classroom?	Developing grouping criteria, spatial arrangement, and creating alternative representations within the context of the play centers in their own classroom. This activity focuses on children’s ability to establish the relationship between organizing data and reorganizing the representation.	Grouping the toys according to their attributes, developing appropriate classification criteria, organizing the data, systematically placing the data cards on the grid, noticing relationships between groups, reorganizing the representations, creating individual graphical representations, reading and interpreting the graph.

**Figure 1:**
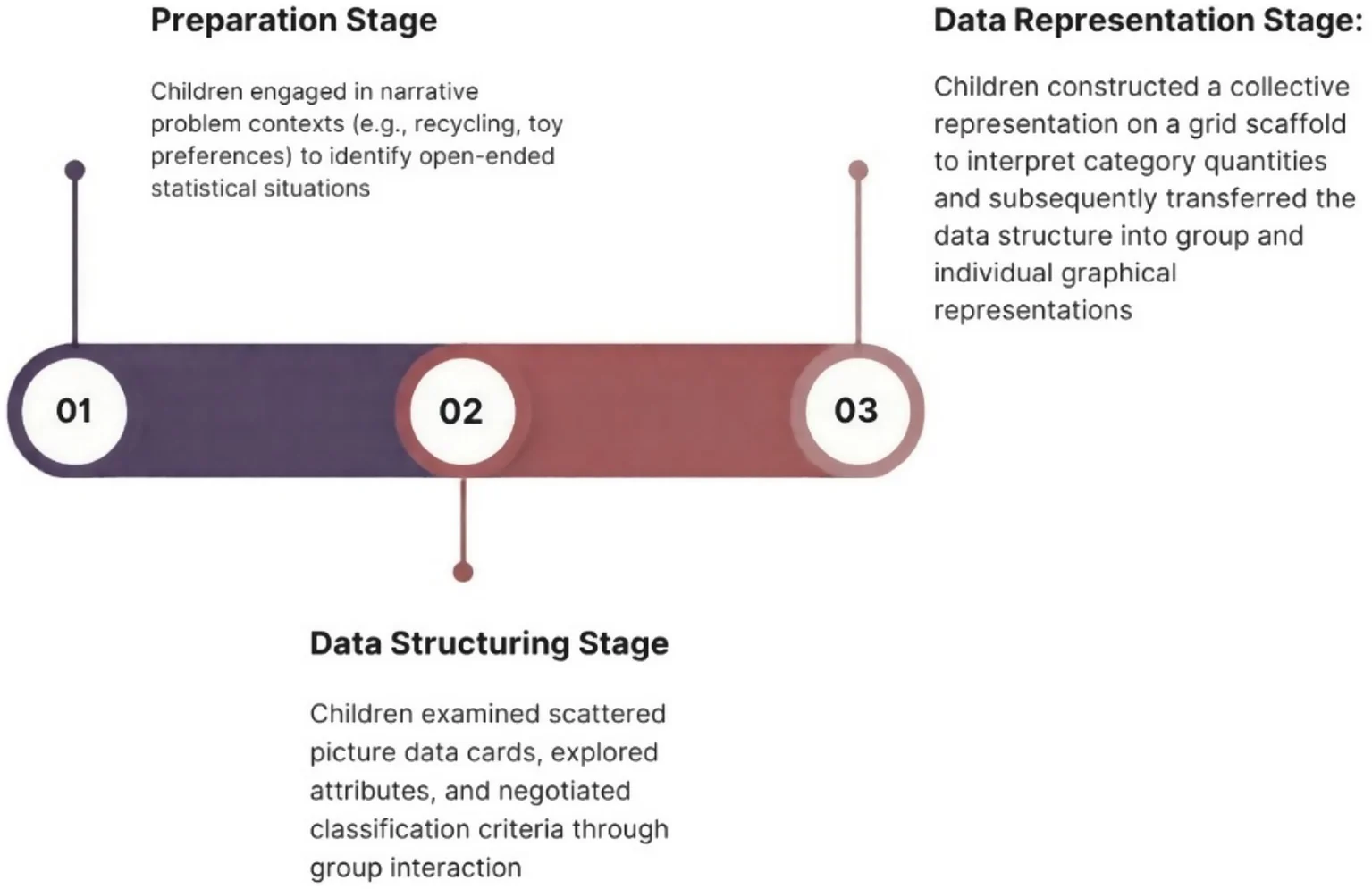
The three sessions of each data modeling activity (the analysis reported in this article focuses on session 2 of the museum activity).

This article analyzes only the museum activity carried out in the second week. All twelve sessions were video recorded. After reviewing the recordings of the four activities, we purposefully selected the museum activity carried out in the second week for the present analysis as an information-rich case (Patton, 2014): it was the activity in which the children’s negotiation of attributes was most visible. Restricting the analysis to a single activity also allowed us to examine the groups’ interactions in fine-grained detail [across all three sessions of the activity] rather than summarizing them across activities. Because we selected the activity after examining the data, the findings are not presented as representative of all four activities.

In that museum activity, the children were presented with an unstructured data set of historical artifacts. They first examined the data cards, then identified the attributes they considered appropriate, constructed a data organization based on the attributes they selected, and discussed their group decisions. Their processes of identifying attributes and structuring emergent variables could thus be observed in detail Figure 2.

**Figure 2:**
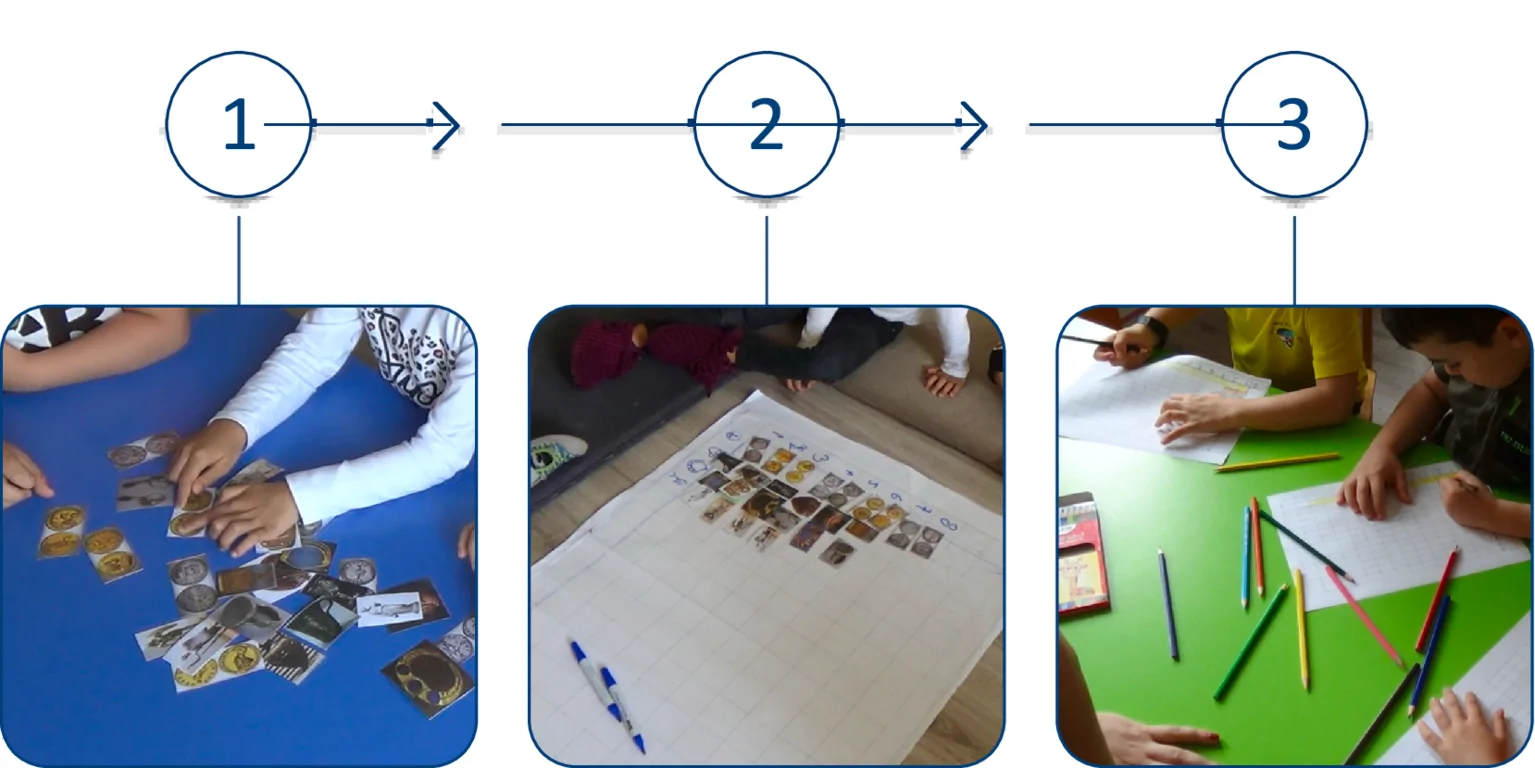
Children’s work across working on the museum activity.

### 2.4 Data collection

The primary data source of the research is video recordings. Each group’s interactions were recorded continuously throughout the group work. The recordings were analyzed in two modalities: the children’s talk was transcribed in full, and stills from the recordings were used to document how the cards were handled and arranged at particular moments. Alongside the video record, the children’s products, the worksheets they completed, and the card arrangements they produced—and researcher observation notes taken during the sessions—served as additional data sources. Bringing together a record of the process (video), the artifacts the children produced, and an independent contemporaneous account (observation notes) allowed the findings to be corroborated from different angles. They provided data triangulation (Lincoln and Guba, 1985). Investigator triangulation was additionally achieved through the two researchers’ independent coding and their subsequent comparison of analyses.

### 2.5 Data analysis

The data were analyzed through a cross-case analysis approach (Stake, 2006), proceeding first by examining each group independently and then by comparing common patterns and diverging decision processes across the groups.

The video analysis followed the qualitative video analysis model proposed by Powell et al. (2003). In the first phase, all recordings were viewed repeatedly, critical events were identified, and group interactions were transcribed in detail. The transcription covered not only speech but also nonverbal behaviors such as pointing, placing cards, and shifts in orientation.

The coding process was carried out with reference to the cyclical coding approach proposed by Saldaña (2021). In the first cycle, all children’s statements were identified through open coding; in the second cycle, similar codes were grouped into higher-order conceptual categories. Throughout, codes were continually compared with previously analyzed data, redefined when necessary, and developed as the analysis progressed (Miles et al., 2020; Saldaña, 2021).

Four main categories explaining the children’s data modeling process were formed at the end of the analysis: (1) noticing attributes, (2) selecting attributes, (3) attribute structuring and emergent variable construction, and (4) measuring attributes.

The coding included not only the children’s successful data organization but also their attribute revisions, hesitations in selecting categories, disagreements within the group, and unsuccessful attempts, so the data modeling process was examined in a process-oriented rather than a product-oriented way.

The activities, the children’s talk, and all transcripts were produced in Turkish. The first author translated the excerpts presented in this article into English, and the second author checked the translations against the Turkish transcripts. Because the children’s speech was elliptical and grammatically irregular, the translations preserve that irregularity rather than rendering it in fluent adult English; material added for comprehensibility appears in square brackets.

### 2.6 Trustworthiness and ethics

To strengthen the research’s trustworthiness, the researchers considered the principles of credibility, transferability, dependability, and confirmability proposed by Lincoln and Guba (1985). To achieve triangulation, the researchers evaluated video recordings, researcher observation notes, and children’s products together. Throughout the coding process, the researchers compared their analyses and reached agreement by discussing divergent interpretations. The researchers also included children’s own words in the findings and supported interpretations with data to strengthen credibility (Lincoln and Guba, 1985). The coding and theme development phases also followed the constant comparison and analytic memo-writing approach proposed by Saldaña (2021), and the researchers reviewed the categories regularly to ensure they addressed the research questions.

Before the research began, the Human Research Ethics Committee of Sinop University granted the necessary ethical approval, and the Provincial Directorate of National Education granted permission to implement the study. The researchers obtained written informed consent from the parents of all participating children and kept confidential any information that could reveal the children’s identities throughout the research process.

## 3 Findings

This section presents the cross-case analysis findings on the two groups’ data-modeling processes during the museum activity. The analysis identified four categories: noticing attributes, selecting attributes, attribute structuring and emergent variable construction, and measuring attributes. Each category is first described within the groups, followed by a cross-case evaluation. The data for each category are described through direct quotations selected from the children’s talk; quotations are presented in italics and, where necessary to explain how a decision took shape, are accompanied by nonverbal behaviors such as placing a card or pointing.

### 3.1 Noticing attributes

A comparison of the two groups’ data modeling processes shows that the children systematically described the objects’ salient properties before organizing the unstructured data set. They did not merely name the data cards; they also voiced different attributes of the same object one after another. Noticing attributes took four forms: (a) noticing object type, (b) noticing perceptual attributes, (c) noticing attributes relating to material, and (d) noticing more than one attribute simultaneously on the same object.

#### 3.1.1 Noticing attributes based on object type

In both groups, the first attribute the children attended to was the type of the objects on the data cards. When they began to examine the data set, and before making any classification, they described the cards with names such as “coin,” “vase,” “statue,” “crown,” “necklace,” and “ring,” which indicates that they initially perceived the objects in terms of the object type to which they belonged. In the first group, Ayla’s “Coins,” Hasan’s “Rings,” and Ahmet’s “Vase” and “Necklace” show that they distinguished the objects according to their types; similarly, in the second group, Can said “King’s crown” and Ali said, “Coins… rings, necklaces, crowns, vases,” describing the data set directly in terms of object types. On first encountering the data set, the children in both groups focused on the objects’ type names: the first reading of the data cards rested on recognizing the objects, prior to considering functional or perceptual attributes.

#### 3.1.2 Noticing perceptual attributes

After naming the objects, attention in both groups turned to directly observable properties, and color was the attribute the children mentioned most frequently. In the first group, Hasan’s “There are gold-colored ones, there are silver-colored ones, and there are brown ones too” shows that he had begun rereading the data set by color. Ahmet accounted for the same data set with different color categories, saying “There are gold color ones, there are silver color ones, and there are dark color ones too.” A similar pattern appears in the second group: in response to the researcher’s question, “What colors is it made up of?,” Buse answered “Gold!” and Ali added the other colors, saying “Brown and silver colors,” after which the group began to separate the data cards into three color groups. The two groups have one thing in common: the color attribute was not supplied directly by the researcher but was selected by the children from the data set and thereby made visible. In the first group, however, the color proposal emerged after a function-based classification, whereas in the second group color became a shared classification criterion at a much earlier stage.

#### 3.1.3 Noticing attributes relating to material

The children did not confine themselves to directly visible properties; they also inferred what material the objects might be made of, indicating that an attribute not explicitly written on the data cards was identified based on visual cues. In the first group, Ahmet said, “This one’s an iron coin!” while Ayla said, “This is a gold coin then,” attempting to describe the objects by the material they were made of. Similarly, in the second group, after the researcher asked, “What is it made of?,” Ali said, “Out of gold,” Can said, “This statue is made out of iron,” and Buse said, “Turns out there is a concrete statue too.” Can said, “I found a silver cup too,” showing that the children treated material as an independent attribute. Although both groups noticed material, they did not sustain it as a classification criterion in the subsequent data-organization process: the children could describe material but preferred the more salient attributes of color or object type when organizing the data.

#### 3.1.4 Noticing more than one attribute on the same object

Another notable finding is that the children described the same object not by a single attribute but by multiple attributes, and the coin cards provided clear examples of this. In the first group, the same object was described first as a “coin,” then as a “gold coin,” then as a “silver coin,” and finally as an “iron coin.” Ahmet’s “If it were a coin, Atatürk would be on it” [Atatürk’s portrait appears on Turkish coins] shows that he attended not only to the object’s appearance but also to its functional properties. Similarly, the children in the second group described the same object in terms of both type and material, using expressions such as “There is an iron coin too, there is a gold coin too,” “There is a gold crown,” and “There is a ring out of iron.” These examples show that the children did not perceive the elements of the data set in one dimension; they noticed multiple attributes simultaneously on the same data card.

#### 3.1.5 Cross-case analysis

Comparing the two groups reveals that the process of noticing attributes followed a similar developmental pattern. Both groups first described the data set in terms of object types, then turned to perceptual and material properties, and for some objects voiced more than one attribute at the same time. An important difference was nonetheless observed. In the first group, children discussed different attributes together over an extended period and moved back and forth between function and color; in the second group, they noticed material and color earlier and quickly turned them into a shared classification criterion. This finding shows that the children identified potential attributes in the data set before organizing it, but these attributes took shape through within-group discussion. Noticing attributes can therefore be regarded not merely as an observational phase in the data modeling process but as a cognitive starting point that prepares subsequent attribute selection and category construction.

### 3.2 Selecting attributes

After noticing the attributes, the children in both groups were seen deciding on the criterion by which to organize their data. In this process, they did not use all the attributes they had observed in the dataset; instead, they selected the attributes they found meaningful for solving the problem as classification criteria. The cross-case analysis shows that this selection followed two basic patterns: (a) conceptual/contextual attribute selection based on object type, and (b) perceptual attribute selection. Notable differences were nonetheless identified between the groups regarding which of the two approaches predominated.

#### 3.2.1 Conceptual/contextual attribute selection

In the first group, the initial classification proposals were developed based on the objects’ function or type. In response to the researcher’s question, “How would you like to classify them?,” Ayla answered, “Coins with coins, vases with vases, necklaces with necklaces,” indicating that she used object type as the primary criterion for organizing the data. During the same discussion, the researcher’s summary of the children’s proposals, “you said ‘I will separate the vases, the jewelry, the coins, the statues’“—likewise indicates that the group’s first shared decision was based on object type. Later, as the work was divided, Ayla’s “let me separate the coins,” Hasan’s “me the jewelry,” and Ahmet’s “me the statues” show that the group agreed on the criterion and organized the data accordingly.

In the second group, the process began differently. The children voiced object types while examining the data cards, in response to the researcher’s question, “Which attribute are you thinking of grouping them by?” Can answered directly, “colors!” Following this proposal, Ali added the other colors, saying “brown and silver,” and the group quickly developed a shared color-based organization. This finding shows that although both groups noticed object type, they did not select the same attribute at the data-organization stage. In the first group, object type served as the organizing criterion, whereas in the second group, this attribute was abandoned earlier in favor of color.

#### 3.2.2 Perceptual attribute selection

Perceptual attribute selection occurred in both groups primarily via color. However, the way this selection emerged differed between the groups. In the first group, Ahmet’s “the golds, the silvers…,” Hasan’s “gold silver,” and later “There are gold-colored ones, there are silver-colored ones, and there are brown ones too” show an attempt to reorganize the dataset by color. Ayla, by contrast, tried to preserve object type within the same discussion, saying “they are coins, these!” Later, the researcher’s assessment—"now you have made two different classifications”—also shows that the group was discussing two different organizing criteria simultaneously.

In the second group, members adopted the color proposal more quickly. Can’s proposal “gold!” was completed by Buse’s “brown and silver,” after which the children began to separate the data cards into three color groups. The group members did not discuss an alternative classification proposal at length; color quickly became the shared criterion. This shows that perceptual attributes were a powerful organizing resource for both groups, but that within-group negotiation played a decisive role in the stability of the selection.

#### 3.2.3 Attribute selection shaped through negotiation

The analysis shows that attribute selection was not an individual decision but took shape through discussions among group members. The children in the first group proposed organizing the same dataset by different attributes and exchanged views on which criterion to use. Ahmet’s proposal, “gold coins, silver coins, jewelry…,” for example, combines color and object type in the same utterance. Hasan tried to sustain the color criterion, saying “There are gold-colored ones, there are silver-colored ones…,” while Ayla continued to name the objects as “coins” and “vases.” When the researcher asked them to return to their first proposals, the group once again organized by object type.

In the second group, by contrast, the group reached a shared decision on attribute selection more quickly. Although the children noticed material and object type, they did not use these attributes to organize the data; instead, the group reached a shared decision based on color. This finding shows that attribute selection cannot be explained by which attributes are noticed alone. Although more than one possible attribute is present in the same data set, within their group interactions the children adopt only one of them as the organizing criterion.

#### 3.2.4 Cross-case analysis

Comparing the two groups shows that the children displayed two common tendencies in attribute selection. First, both groups identified more than one potential attribute in the data set at the same time but used only some of these attributes to organize the data. Second, attribute selection took shape through group discussions rather than individual observations.

Nonetheless, an important difference emerged between the groups. In the first group, the group negotiated between object type and color, whereas in the second group, color became the shared organizing criterion early on. This difference shows that attribute selection is influenced not only by the properties of the dataset but also by how group members evaluate one another’s proposals. Attribute selection in the data modeling process can therefore be seen less as determining a single correct criterion than as a joint decision-making process in which the group discusses and provisionally stabilizes alternative forms of organization.

### 3.3 Attribute structuring and emergent variable construction

The cross-case analysis shows that children selecting the attribute they considered appropriate was not sufficient for organizing the data. Applying the selected attribute to the entire dataset, determining category boundaries, and placing each object in the appropriate category required continuous negotiation within the group. In this process, the children sometimes sustained the criterion they had selected and sometimes abandoned criteria they considered inadequate for accounting for the data set, turning to new forms of organization. The analysis shows that this process involves four basic patterns: (a) determining category membership, (b) noticing category–object mismatches, (c) revising the categorization criterion, and (d) justifying the category selection.

#### 3.3.1 Determining category membership

In both groups, as they applied the attribute they had selected to the dataset, the children sought to reach shared decisions about which category each object belonged to. These decisions were usually not made by a single child but took shape through the group members’ reciprocal proposals.

In the first group, after deciding to classify by object type, the children divided the work and began organizing the data set. Ayla’s “let me separate the coins,” Hasan’s “me the jewelry,” and Ahmet’s “me the statues” show that the group jointly accepted the category boundaries. Ayla then separated the coins one by one, and Hasan said, “I will put the jewelry,” revealing that the categories they had determined were beginning to be used to organize the data Figure 3.

**Figure 3:**
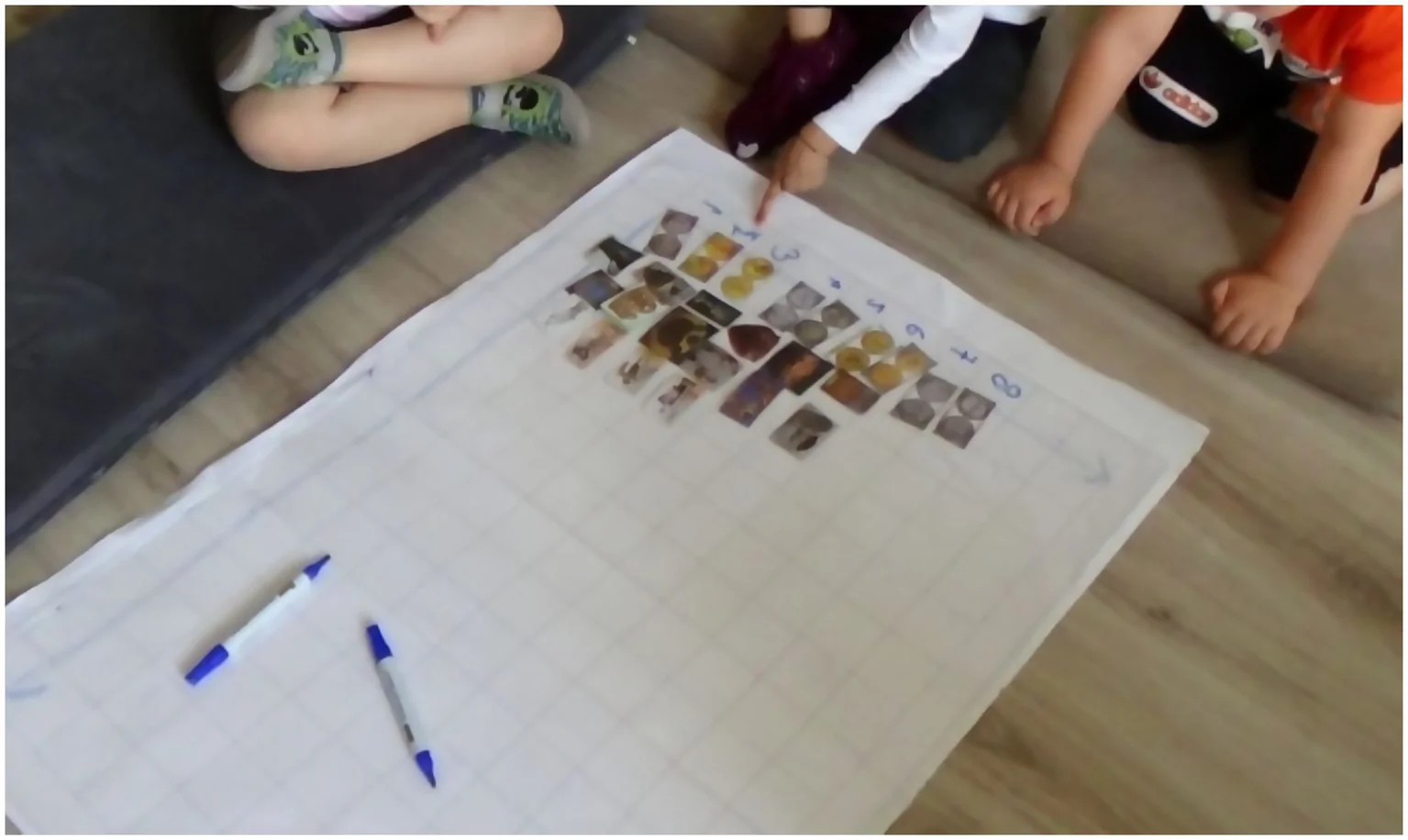
The first focal group’s classification of the data cards by object type.

In the second group, they formed category membership based on color. In a process that began with Can’s proposal “gold!,” the other category values were determined by Ali’s “brown and silver,” and the children placed the cards into these three-color groups. These examples show that forming categories is not limited to identifying an attribute; it requires systematically applying the selected attribute to the whole data set Figure 4.

**Figure 4:**
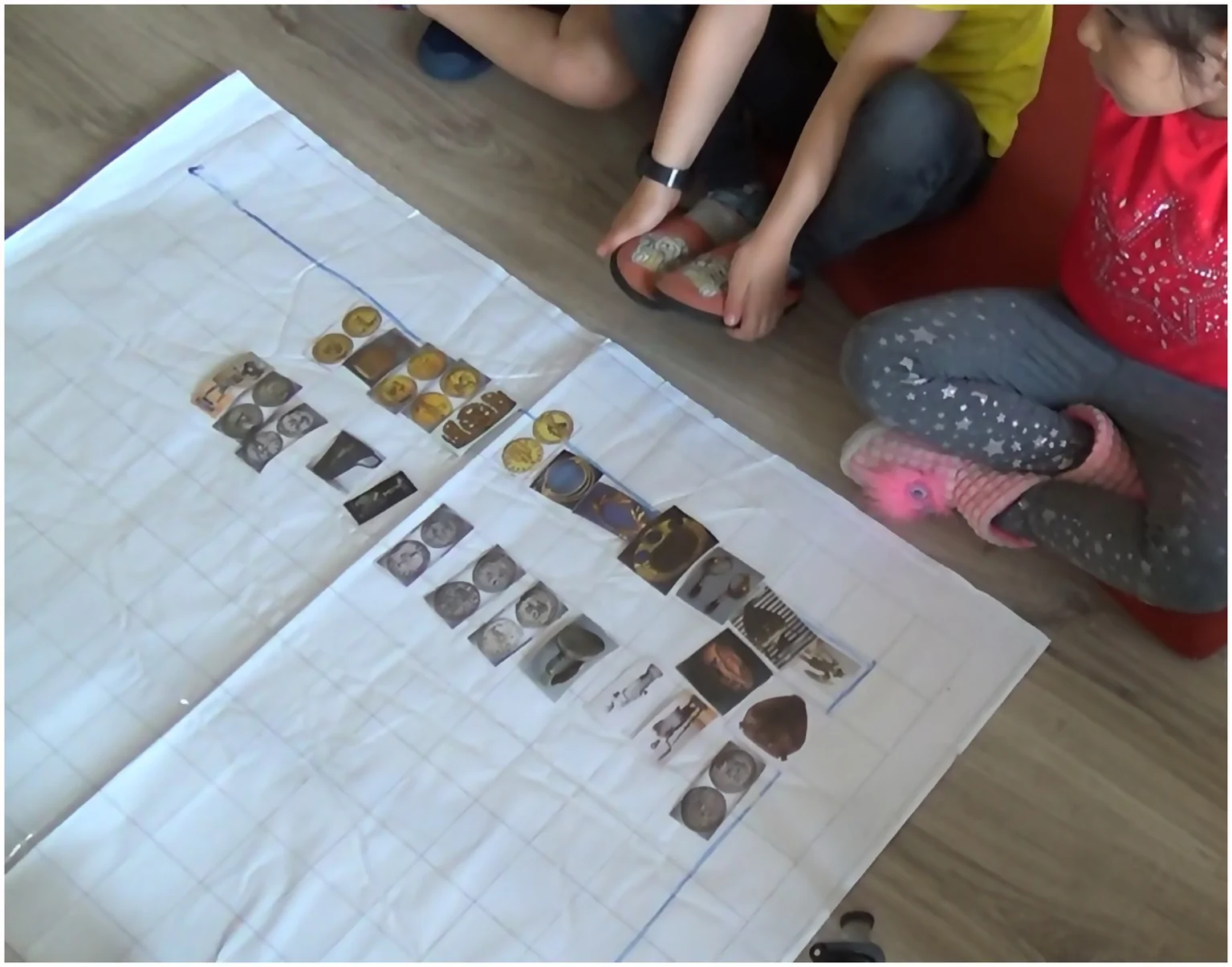
The second focal group’s classification of the data cards by color.

#### 3.3.2 Noticing category–object mismatches

During data organization, the children noticed that some objects did not fully match the categories they had formed. This became particularly apparent where the same object possessed more than one attribute. In the first group, Ayla asked, “Teacher, is not this a coin?” and Ahmet answered, “If it were a coin, Atatürk would be on it,” showing that they evaluated an object not only by its appearance but also by the properties it was expected to have. After this discussion, Ayla’s saying “this is a gold coin then” reveals that the group reinterpreted the object’s category membership.

Similarly, in the second group, the children initially received the expression “Concrete statue” with surprise; after Buse’s question, “Concrete?,” Ali showed the card again and said, “Yes, look,” and the group reviewed its initial assessment of the object’s material. These examples show that the children did not determine category memberships mechanically; they questioned the fit between category and object and, when necessary, reconsidered their initial decisions.

#### 3.3.3 Revising the categorization criterion

One of the most striking findings in the cross-case analysis is that, during data organization, the children did not always sustain the criterion they had adopted at the outset. This was especially clear in the first group. In the first part of the activity, Ayla proposed organizing by object type, while Hasan proposed organizing the same data set by color. Although Hasan later developed the color criterion, “There are gold-colored ones, there are silver-colored ones, and there are brown ones too,” the group once again turned to an organization based on object type when the researcher asked them to return to their first proposals Figure 5.

**Figure 5:**
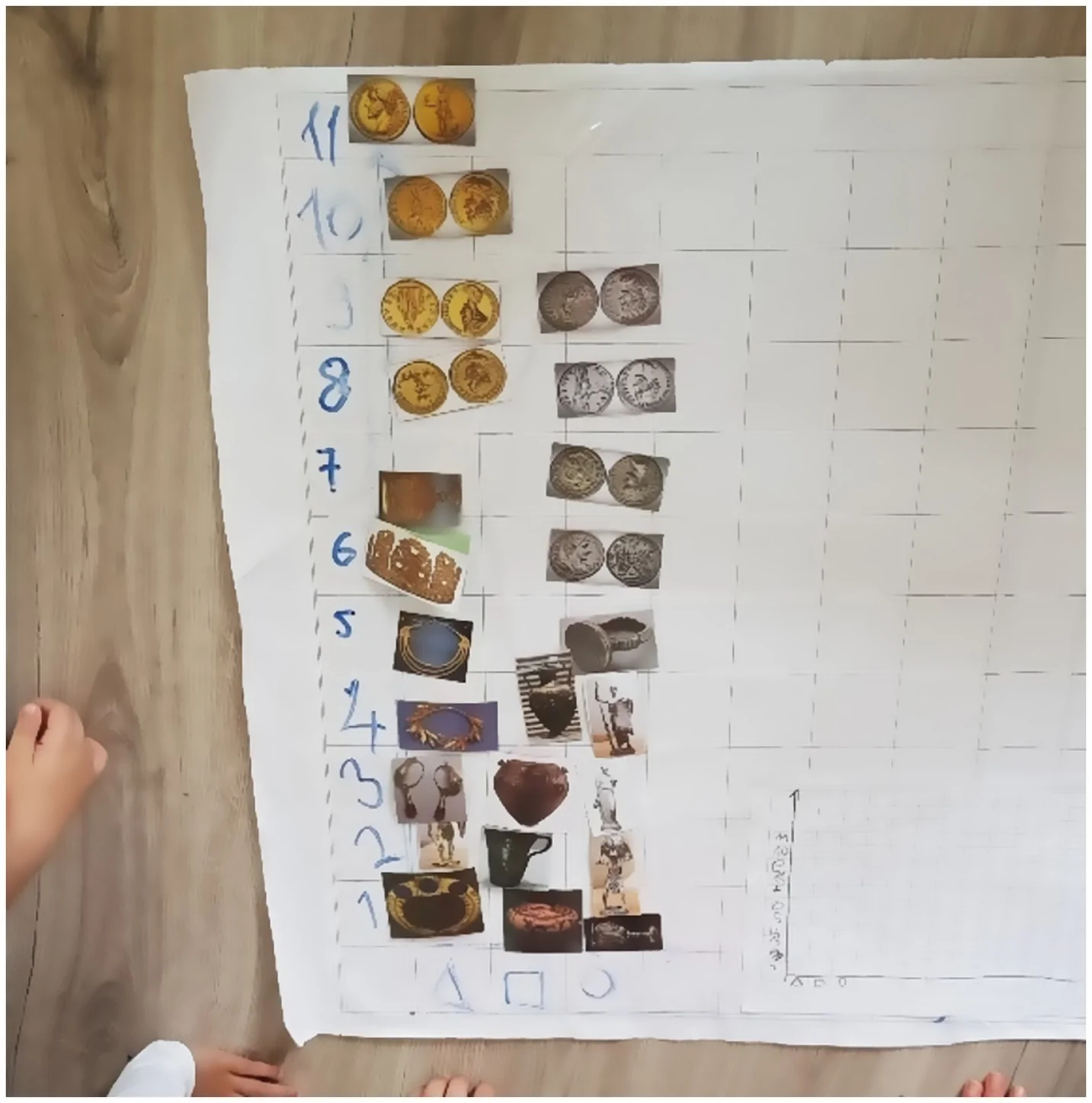
The first focal group’s revision of the classification criterion: reorganizing the data cards by color.

Notably, the children did not revise the criteria arbitrarily. The criterion revision emerged from the group’s discussion of different organizational proposals. The revision therefore indicates not that the previous criterion was entirely wrong, but that the children had recognized that the same data set could also be organized with different attributes.

No similar revision of the criterion was observed in the second group. Although the children voiced material properties, they preserved the color criterion throughout the data organization and did not develop an alternative organization. This difference shows that the groups managed the data organization process differently. In one group, they discussed alternative criteria for an extended period, whereas in the other, the shared criterion stabilized earlier.

#### 3.3.4 Justifying the category selection

In some cases, the children not only applied the categories they had formed but also attempted to explain why they placed an object in each category. These explanations are important because they make the reasoning behind the category decisions visible.

In the first group, Ahmet’s explanation, “if it were a coin, Atatürk would be on it,” shows that he linked his category decision to the object’s expected functional properties. Similarly, Ayla’s “There are gold-colored ones, there are silver-colored ones” shows that she based the categories she formed on a shared property in the data set. In the second group, the explanations focused more on defining the category values. Following Can’s proposal, “colors!,” Ali’s “brown and silver” established a shared framework for applying the selected criterion.

These explanations show that, in forming categories, the children did not merely place cards; they also attempted to explain why the organization they selected was appropriate. The level of detail in the explanations nonetheless varies by group and by context.

#### 3.3.5 Cross-case analysis

Comparing the two groups shows that attribute structuring did not follow a linear progression. Both groups attempted to turn the attribute they had selected into a data organization. However, in doing so, they discussed category memberships, reconsidered the placement of some objects in the appropriate category, and negotiated the organization they had formed within the group. The most marked difference is the criterion’s stability. In the first group, the data organization developed as a dynamic process moving back and forth among alternative attributes. In contrast, in the second group, the selected criterion stabilized earlier, and the organization was sustained around that criterion. This finding shows that constructing a variable in early childhood does not consist merely of identifying a single attribute; it is a dynamic process that develops through the formation of category boundaries, the continual evaluation of the object–category relation, and group agreement on a shared data structure.

### 3.4 Measuring attributes

After identifying the attributes and completing the data organization, the children in both groups turned to determining the sizes of the categories they had formed. The analysis shows that the children used three different strategies to determine these group sizes: (a) direct counting, (b) reading from the representation they had constructed, and (c) using counting and reading together. These strategies show that the children were not content merely to form categories but also sought to evaluate the data structure they had constructed quantitatively.

#### 3.4.1 Direct counting

In both groups, the most common measurement strategy was to count the objects in a category one by one. To determine how many elements each category contained, the children counted systematically, pointing to the objects.

In the first group, after placing the coins, Ayla counted the objects in order, saying “One, two, three, four, five, six, seven, eight, nine,” which shows that she determined the group size directly from the objects. During the counting, each number was matched with a specific object, and the group members waited for the count to finish without intervening. Similarly, in the second group, after forming the color groups, the children checked the cards one by one and counted the objects belonging to each category. The group members shared the counting; the children followed the cards within the same category together and arrived at a shared result. This finding shows that in both groups the process of determining quantity was carried out directly on the data objects.

#### 3.4.2 Measuring from the representation

In some cases, the children began to interpret the data organization they had constructed not only through the objects but also through the representation they had prepared. The representation was no longer merely an arrangement in which the objects were placed; it had become a reference from which they could read category sizes.

In the first group (Excerpt 1), after the children began constructing the graph, they evaluated the heights of the category columns and the numerical expressions placed beneath them. The group members were able to compare, through the representation, that categories had more or fewer elements. This shows that the children could read the organization they had constructed not only from the physical objects but also from the structure of the representation.In the second group (Excerpt 2), although expressions relating to measuring from the representation were more limited, the children checked the size of each group after forming the color groups. They used the arrangement of the representation as a tool supporting the counting.This finding shows that, for the children, the representation had begun to function not merely as a form of display but as a tool for rereading quantitative information.

Excerpt 1 Researcher: How many were the jewelry ones?

Ayla: Four

Researcher: Where is the jewelry's place?

Ayla: [pointing with her hand] Here

Researcher: Why?

Ayla: There's the jewelry's mark, its symbol… the jewelry and the vases are equal in number

Researcher: Children, your friend Ayla says the jewelry and the vases are equal in number. Is she right?

Hasan: Yes, look, the same

Researcher: Yes. And which is there the most of?

Ahmet and Hasan: The coins

Researcher: Which is there the least of?

Ahmet: Statue

Researcher: How could you tell?

Hasan: Because the number over there is small.

Excerpt 2 Researcher: And how many more are the silver ones than the brown?

Can: Ten!

Ali: Ten

Researcher: How did you do it?

Ali: By counting!

Researcher: What did you count?

Ali: The boxes. I counted the missing boxes.

Researcher: The missing boxes?

Ali: I counted the boxes that weren't colored in. If we had colored them, ten more, then they'd be equal, the same height I mean!

#### 3.4.3 Using counting and the representation together

A notable point in the cross-case analysis is that the children used direct counting and reading from the representation together. The children first counted the objects in a category one by one, then re-examined the arrangement they had constructed to check the result.

In the first group (Excerpt 3), Ayla’s first counting the objects one by one and then reviewing the placement on the graph provides an example of this. The counting served not only to obtain the result but also to check the accuracy of the representation.

Excerpt 3 Researcher: All right, now, can we read this graph, children? For example, how am I going to tell how many statues there are?

Hasan: Where the statues end, what number it says across from it

All: Four

Researcher: Yes!

Ayla: But let us count too. One, two, three, four!

Researcher: So there really are four, aren’t there?

All: Yes

Researcher: Then let us look at the coins—how many do you think there are?

Group: Eight! [pointing to the top of the column].

Similarly, in the second group (Excerpt 4) the children re-examined the color groups they had formed, checked the category sizes, and, when necessary, reorganized the placement of the cards. In this process, the children used representation and direct counting as complementary strategies Figure 6.

**Figure 6:**
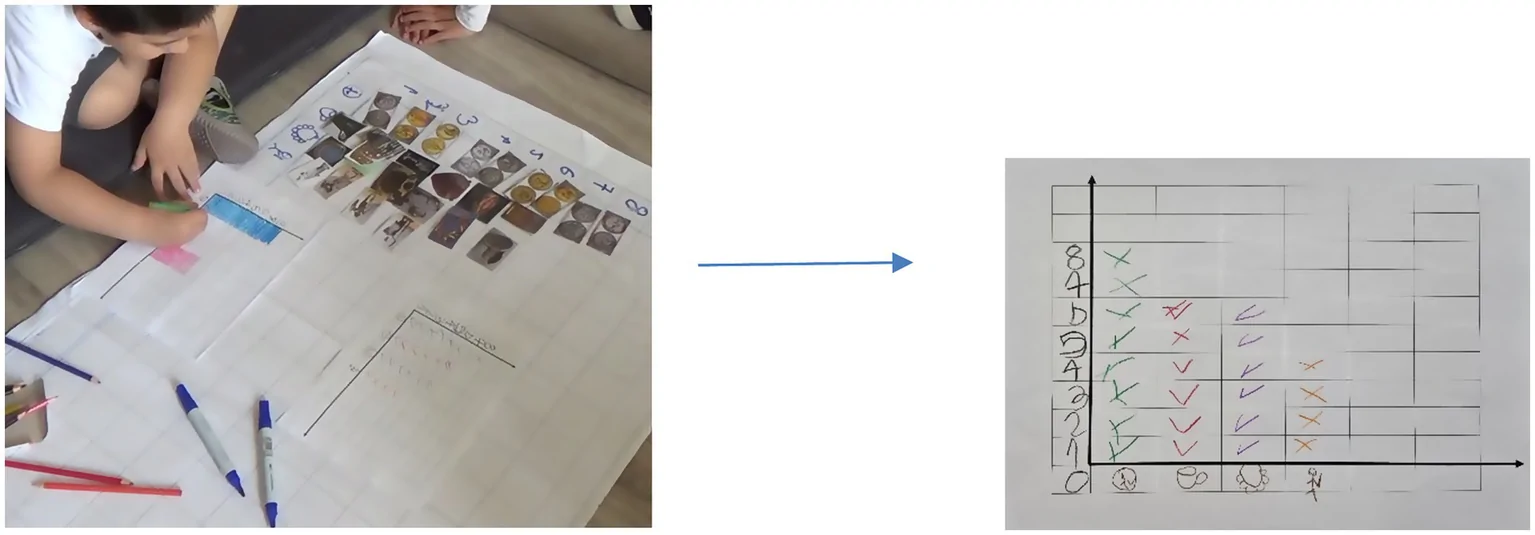
From grid to graph representation (Group 1).

Excerpt 4 Excerpt 1Researcher: How many historical artifacts are there among the gold ones?

[Can]: Twelve!

Researcher: How did you decide there are eleven? Why did not you count them one by one?

[Can]: Because there are numbers underneath it. I looked at the numbers underneath.

Researcher: And how many of the brown objects are there?

[Can]: Two!

Researcher: How did you decide on the number?

[Can]: Because these ended at two.

These examples show that the measurement process was not merely a mechanical counting activity; the children used multiple sources of information to evaluate the accuracy of the data organization Figure 7.

**Figure 7:**
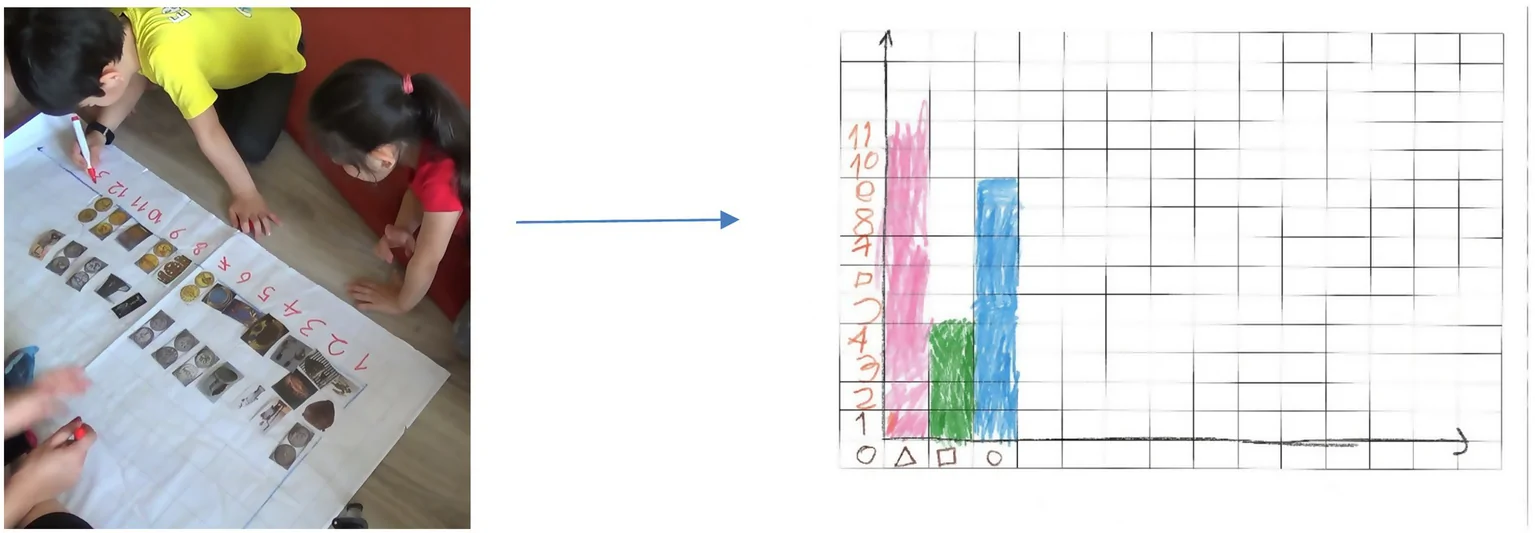
From grid to graph representation (group 2).

#### 3.4.4 Cross-case analysis

Comparing the two groups reveals a common developmental pattern in the measurement of attributes. After completing the data organization, both groups felt the need to determine category sizes and first used direct counting. As they constructed the representation, however, the children’s attention shifted from individual objects to the representation. The first group read quantitative information from the representation more clearly, whereas the second group used the representation more as a tool to support direct counting.

This finding shows that although measuring attributes is the final phase of the data modeling process, it is not independent of the preceding decision processes. The categories the children formed and how they structured them directly affected the quantitative evaluations they subsequently conducted. The measurement process, therefore, does not consist merely of determining group membership; it can be regarded as a holistic data-modeling activity that the children used to verify, compare, and reconsider the data structure they had constructed.

## 4 Discussion

This study examined which attributes preschool children noticed while working with an unstructured data set, which they selected for organizing the data, and how they turned those attributes into category structures. Taken as a whole, the findings indicate that constructing a variable in the data modeling process is not a single-step decision in which children notice an attribute and use it directly as a classification criterion; rather, variable construction becomes progressively more definite within the processes of noticing the attributes of data units, negotiating possible criteria, agreeing on a criterion, forming category values, and testing whether the resulting structure works on the data set.

This result aligns with approaches that treat statistical thinking not merely as performing operations on data or producing results, but as a process of deciding what counts as data worth attending to and which attribute the data will be examined with respect to (Garfield and Ben-Zvi, 2008; Wild and Pfannkuch, 1999). It also supports the GAISE II position that, in early statistical investigations, children should establish relations among problems, data, analysis, and interpretation through their active decisions (Bargagliotti et al., 2020).

### 4.1 Noticing attributes

The first research question focused on which attributes children identify as potential classification criteria in an unstructured dataset. That children in both groups could name more than one attribute—object type, color, and material—on the same data cards shows they did not reduce the data set to a single dimension. This finding matters because, in early thinking with data, children must first see which differences are statistically usable. The result also relates to Lehrer and Schauble's (2004) position that variables are not structures already present in a data set and merely waiting to be discovered but are structured in line with the problem and the purpose of the inquiry. The important distinction here is between noticing an attribute and structuring that attribute as a variable. This study’s findings take this approach one step further: children noticing an attribute does not mean the attribute immediately becomes a variable. Voicing differences in object type, for example, indicates that a difference among the objects in terms of type has been noticed, whereas using “object type” as a shared criterion to organize the whole data set requires a further degree of structuring. Noticing an attribute can therefore be thought of less as variable construction itself than as an initial process that makes it possible for potential variables to emerge.

This interpretation is also meaningful in relation to the literature on variation. Wild and Pfannkuch (1999) emphasize that attending to variability lies at the basis of statistical thinking, while Lehrer and English (2018) point to the importance of thinking from individual objects toward the data as an aggregate when children make sense of variation. The results of the present study are nonetheless insufficient to conclude that the children possessed a formal understanding of distribution or variation. More modestly, the children could notice which attribute the data units could differ on. This distinction matters because, beyond merely seeing objects as different, determining which attribute a difference is tied to forms the basis of subsequent data organization.

Buehring and Grando's (2023) findings that young children can work with data situations involving more than one variable are also meaningful here. In the present study, the children’s identification of multiple potential dimensions—type, color, and material—within the same data unit can be seen as an early indicator that a data set can be structured in different ways. For example, associating the same object with “coin,” “gold,” and a particular material indicates that the data unit was not seen as one-dimensional. It would nonetheless be inappropriate to call this finding multivariate statistical thinking; it is better described as an awareness of multiple attributes that may support multivariate thinking at later stages. Kurt (2023) results, showing that young children can display more complex statistical and probabilistic reasoning than expected when appropriate contexts are provided, also support this interpretation. The present study suggests that such complexity may be visible not only in situations of inference or uncertainty, but also at the very early stage when children decide which attributes to consider before data analysis begins. In other words, children’s thinking with data does not begin only with answering the question “how many?”; it may emerge earlier, with the formulation of the question “the same or different in what respect?”

### 4.2 Attribute selection

The second research question concerns which attributes the children noticed and selected, and how they justified those selections. An important finding is that the noticed attribute and the selected attribute are not the same. Although both groups noticed color, material, and object type, not all of these attributes became criteria that guided the data organization. The first group’s longer movement between object type and color, and the second group’s quicker agreement on color, show that attribute selection is not derived solely from the visual properties of the data cards. This result is closely related to Leavy and Hourigan (2018) findings that, alongside perceptual similarity, context and situation also influence 5 to 6-year-old children’s attribute selections. Children do not always rely on the most visible attribute; they can turn to other criteria based on their knowledge of the problem and the objects. The present study extends this result to group interaction: children’s decisions about which attribute to use were shaped not only by individual perception or contextual knowledge but also by encountering and evaluating their peers’ alternative proposals.

The role of context is also consistent with Kinnear (2023) study, which shows that data context and task context influence young children’s interpretations of data representations. Frischemeier and Schnell (2023) show that children draw on contextual expectations during data analysis, and Zapata-Cardona (2023) shows that in early data modeling, meaningful, culturally relatable contexts can affect the quality of reasoning with data. Considered together with these studies, the classification proposals in the present research — “coin,” “jewelry,” “gold,” “silver,” or “material”—may be taken not to arise from a visual reading of the cards alone. The children’s prior knowledge of the historical artifact context and their expectations about the objects also appear to have influenced which attribute was accepted as meaningful. At this point, it is important not to place perceptual and conceptual/contextual attributes in a simple developmental hierarchy: selecting color is not necessarily an indicator of lower-level thinking, nor is selecting object type an indicator of more advanced thinking. The quality of an attribute selection must be evaluated alongside the selected attribute’s relevance to the problem context, its applicability across the entire data set, and whether the children were able to construct a consistent data structure using that criterion. This interpretation also aligns with contemporary statistical modeling, which treats data not as quantities detached from context but as relations among problem, data, and meaning (Noll et al., 2022).

A second important dimension of the findings is the social character of the attribute selection process. In the first group in particular, the coexistence, for a time, of alternative proposals for organizing the same data set by color and by object type suggests that the children were not merely voicing preferences formed in advance but were making reciprocal evaluations of which criterion to use. The findings therefore show that negotiation occurs before and during attribute selection. Fry et al. (2024) “cognitive tuning” findings, which show that classroom communication mediates changes in children’s understandings of data, support this interpretation. The present study shows that communication can function not only in interpreting already constructed data structures but also earlier, in determining which attribute will form the basis for data organization. Social interaction thus appears less as an auxiliary process to data modeling than as a mechanism that directly participates in constructing the data structure.

### 4.3 Structuring the selected attribute

The third research question focused on how children formed, negotiated, and, when necessary, revised category structures as they applied the selected attribute to the data set. The findings show that attribute selection does not automatically produce data organization. The children had to decide which values the selected attribute could take, position each data unit according to those values, discuss uncertain memberships, and test whether the structure they formed worked across the entire data set. This process constitutes the study’s strongest finding regarding emergent variable structuring.

From the perspective of Lehrer and Schauble (2004) approach, constructing a variable requires more than naming an attribute: the attribute must vary across data units and serve as a consistent dimension that enables comparison. The present findings offer a more detailed view of how this can emerge in young children’s group interactions. Children noticing color is one thing; turning “color” into a shared criterion, forming the values “gold–silver–brown,” and arranging all the cards according to that structure is another. Similarly, noting the names of objects becomes an organizing criterion only when “object type” is applied systematically across the entire dataset.

Dvir and Ben-Zvi (2023) treatment of informal statistical models as structures of meaning formed between data and context and revised when necessary is important for explaining this dynamic quality. The present study shows that a similar iterative process can also emerge during five-year-old children’s data organization. In the first group, trying alternative criteria and reconsidering initial classification decisions indicate that participants evaluated the applicability of the structure they formed to the data set during the process.

This is also consistent with the iterative understanding of modeling emphasized in English (2010, 2012, 2013, 2014) work on early data modeling. In English’s studies, children move nonlinearly among understanding the problem, identifying variables, organizing the data, and reevaluating the structures they have developed. The results of the present study show how this iterative movement can occur specifically in the process by which an attribute becomes a variable. Children’s turning from one criterion to another should therefore not be read merely as indecision or error.

Caution is nonetheless required here. It is not possible, with the present data, to claim that every revision of the criterion was a deliberate act of model evaluation. On a more measured reading, revising the criterion may indicate that the children were reevaluating the adequacy of the data structure they had formed. This interpretation is consistent with Dvir and Ben-Zvi (2023) approach to model revision and with Noll et al.'s (2022) conceptualization of statistical modeling as an ongoing interaction among data, context, and uncertainty.

Mulligan et al. (2024) findings on the pedagogical cycle that connects problem posing, data generation, data organization, and interpretation in the first years of primary school likewise show that data modeling is not a set of fragmented operations. In the present research, the children’s discussion of category memberships and revision of their criteria when necessary suggests that data organization is not a passive intermediate step in the modeling cycle but a productive phase in which the problem and the data are reinterpreted.

Explaining category membership is likewise important for this process. Physically placing a card in a particular category and stating why it belongs there are not the same kind of evidence. The moments in which the children justified category membership made visible which attributes they used to determine category boundaries. It therefore appears more appropriate to understand emergent variable structuring not from classification behavior alone, but through the joint examination of children’s verbal justifications, their card placements, and their responses to peers’ objections.

### 4.4 Variation, category structure, and the quantitative dimension of data organization

In the theoretical framework, variation was not treated as synonymous with classification, but rather as the recognition that data units differ on particular attributes and the organization of those differences into a meaningful structure. The findings support this distinction. The children could name different colors or materials, but they turned only some of the differences into systematic categories. For early forms of variation, the critical point is not merely to notice difference but to determine which attribute the difference occurs on and to apply that attribute to the data as an aggregate (Lehrer and English, 2018; Reading and Shaughnessy, 2004).

Determining category sizes likewise depends on the preceding attribute decisions. Before the children can count how many data units a category contains, category membership must first become stable. GAISE II’s treatment of statistical investigation as a holistic process involving connections among question, data, analysis, and interpretation accounts for this relation (Bargagliotti et al., 2020). In the present study, quantitative evaluation was used to assess the previously formed category structure and to make the groups comparable. Because the study’s main research questions focus on attribute and variable structuring, however, this quantitative process should be evaluated here not as an independent representational competence but as a continuation of the data structure that was formed.

## 5 Limitations of the study

The findings of this study also yield some implications for teaching data in early childhood. Activities in which categories and variables are given to children in advance may be valuable for counting, ordering, or using ready-made data structures; in such tasks, however, the decision about “which attribute matters for the data organization” has already been made on the child’s behalf. By contrast, open-ended data modeling tasks that allow more than one reasonable organization can lead children to identify which attributes could be used, compare alternatives, and test how their selections work on the data set.

Zapata-Cardona (2023) findings on contextual tasks, Mulligan et al. (2024) on holistic data modeling cycles, and Fry et al. (2024) on the importance of classroom communication support this pedagogical interpretation. In early data activities, therefore, the teacher’s role may be not only to confirm a correct classification but to formulate questions that make children’s attribute decisions visible. Questions such as “By which other attribute could we separate them?” “Why is this object here?” “Does this criterion work for all the cards?,” or “If we choose a different criterion, will our groups change?” can support children in reflecting on how the data structure is constructed rather than applying ready-made categories.

The scope of the findings must nonetheless be evaluated with care. The research is limited to the interactions of two focal groups during a particular data modeling task in a single classroom. Moreover, the fact that one of the researchers was the classroom teacher, and that some researcher questions may have directed the children’s attention toward attributes, limits the extent to which the observed process can be interpreted as children’s thinking entirely free of intervention. The account of attribute structuring proposed here should therefore be seen not as a generalizable developmental sequence but as an empirical case arising in a particular natural classroom context. Studies with different age groups, data types, groups, and levels of teacher guidance may reveal which components of this process are context-specific, and which are more stable.

In conclusion, the present study yields a more specific result than a further demonstration that five-year-old children can classify. The findings show that, before and during the formation of a data organization, children notice potential attributes, evaluate them in the light of the context and their peers’ views, form category boundaries, and, when necessary, reorganize the structure they have constructed. In this respect, emergent variable construction can be thought of as discovering a ready-made variable rather than as a process of attribute structuring that develops within data, context, and social interaction. Testing this conceptualization across different data-modeling contexts may contribute to a more detailed theoretical understanding of how variables emerge in early statistical thinking.

## Data Availability

The raw data supporting the conclusions of this article will be made available by the authors, without undue reservation.
